# Mr. Penkivil, on Digitalis

**Published:** 1800-04

**Authors:** John Penkivil

**Affiliations:** Surgeon. Plymouth Dock


### *To the Editors of the Medical and Phyfical Journal.*

GENTLEMEN,

AMONG the numerous admirers of your valuable publication, I feel a pleafure in acknowledging myfelf one of the moft fincere. The liberal plan upon which it is conducted, the extenfive correfpondence it includes, the hints and improvements in the various branches of medical fcience it contains, entitle it to the higheft commendations; and whilft every thing of a perfonal and controverfial nature continues to be fo ftudioufly avoided, its refpectability will be preferved undiminifhed, and its fuccefs thus neceffarily enfured. The opportunity now offered of communicating Obfervations and Cafes, (which otherwife the Public would never have known, and which, perhaps practitioners themfelves might either not have thought of or forgotten) is by no means the leaft confiderable advantage accruing from the publication of the Medical Journal. Partial evils, I am aware, may ſometimes ariſe from your being now and then peſtered by the tedious recital of unintereſting caſes; but the good reſulting from the communication of more important ones is incalculable. I know not whether you will conſider the following caſe as intereſting or not; in ſo far as it tends to confirm the favourable opinion of the anti-phthiſical virtues of a medicine lately introduced (or rather revived) into practice,—I mean, the *Digitalis Purpurea*,—it may probably not be deemed unworthy of notice; and, if ſo, I ſhall think myſelf honoured by its inſertion in an early Number.

About the middle of laſt ſummer, I was deſired to viſit Mrs ———, ætat. 33, who had laboured under complaints of the cheſt for a conſiderable time before, and had medical attendance without relief. I found her emaciated, and much reduced in ſtrength; her appetite though occaſionally tolerable, yet in general very much impaired; her nights were reſtleſs, owing to the conſtant flying pains in her cheſt; and the cough, which was diſtreſſing, followed by a copious expectoration evidently of a purulent nature. The night ſweats were profuſe, and ſometimes made her ſo weak, that ſhe could ſcarcely riſe from her bed. The hectic fever marked by morning and evening exacerbations, ſo accurately deſcribed by Cullen, was completely formed; and her pulſe was about 120 in a minute. The uſual treatment was followed; ſhe was deſired to wear flannels throughout, and expreſſed herſelf much more comfortable from their uſe. Expectorants were ordered, which eaſed the cough without making it leſs frequent; opiates adminiſtered at night, and laxative pills occaſionally given, to obviate coſtiveneſs induced by the opium. The morning and evening febrile acceſſions were ſuſpended, and at length removed, by the exhibition of a powder compoſed of nitre and antimony, juſt before the commencement of an attack. This was the only ground it could be ſaid I had gained, after many weeks attendance.—Sometimes, indeed, the patient would revive, and acknowledge herſelf better; though as often would ſhe ſink, and become as ill as ever; for ſtill the night ſweats continued profuſe, and the expectoration was undiminiſhed, amounting to a full pint in twenty-four hours, and frequently mixed with blood. Tired of a plan which was productive of ſuch partial benefit to the patient, and reflected ſo little credit on myſelf, I determined to try, as a dernier reſource, the Digitalis; and as the tincture is preferred in theſe caſes, I was particular in procuring ſome prepared preciſely according to the formula given us by Dr. Maclean, which for convenience of exhibition, and elegance of appearance, pearance, is far preferable to any other preparation I am acquainted with.

As ſhe was in ſuch a weak ſtate, it was neceſſary to obſerve the utmoſt caution; to let the ſyſtem feel the influence of the medicine in the moſt gradual way, ſometimes repeating ſimilar doſes, and at others increaſing them, according to the effects produced, as well as the ſtate and diſpoſition the patient herſelf was in at the time. The following was the courſe purſued, and the hiſtory of ſymptoms on each viſit.

November 11. Cough and expectoration as uſual, bowels regular, tongue and ſkin natural, pulſe 112.

℞. Tinct. Fol. Digital. gt. x. ex lactis cyatho. = 12. A better night than uſual; fancies her appetite better; expectoration as uſual; b. t. and ſk. natural, p. 110. Augeatur doſis Digitalis ad gt. xi. bis in die. = 13. A good night; ſweated much leſs than uſual; appetite improving; free from pain of breaſt; p. 100, b. t. and ſk. natural, cough and expectoration nearly the ſame. Aug. doſis ad gt. xii. bis in die. = 14. An indifferent night; return of pain of breaſt; cough rather troubleſome; expectoration more conſiderable, and ſlightly tinged with blood; little or no ſweating; appetite tolerable; b. t. and ſk. natural, p. 100. Aug. dos. ad gt. xiii. bis in die = 15. A tolerable night, no ſweats, leſs cough, and expectoration leſs copious, without tinge of blood; has obſerved the tincture keeps her bowels regularly open; complains of ſlight vertigo; free from pain; appetite as uſual; t. and ſk. natural, p. 98, and rather fuller. Aug. dos. ad gt. xiv. bis in die. = 16. A good night, cough and expectoration as uſual, ſlightly tinged with blood; vertigo leſs, appetite not quite ſo good; free from pain; b. t. and ſk. natural, p. 96. Aug. dos. ad gt. xv. bis in die. = 17. A tolerable night; complains of ſlight faintneſs; cough and expectoration leſs, though ſlightly tinged with blood; appetite not ſo good; free from ſweats and pain of breaſt; b. t. and ſk. natural, p. 96. Aug. dos. ad gt. xi. ter in die. = 18. A middling night; cough not ſo troubleſome; expectoration without tinge of blood, and of a more favourable appearance; no ſweats; free from pain, but ſlight vertigo; appetite diminiſhes; b. t. and ſk. natural, p. 96. Aug. dos. ad gt. xii. ter in die. = 19. A good night, cough leſs, and expectoration leſs tinged with blood; not ſo giddy; free from pain; appetite bad; b. t. and ſk. natural, p. 96. Aug. dos. ad gt. xiii. ter in die. = 20. A good night, cough leſs, expectoration alſo leſs, but tinged with blood; had conſiderable pain of breaſt; rather more giddy; no ſweats; appetite bad; b. t. and ſk. natural, p. 92. Aug. dos. ad gt. xiv. ter in die. = 21. A poor night, more pain of breaſt, and more vertigo; vomited conſiderably at midnight; night; cough rather lefs, and expectoration by no means fo copious; appetite bad; no fweats; b. t. and fk. natural, p. 90. Aug. dos. ad gt. xv. ter in die. = 22. A tolerable night; no vomiting; no fweats; cough not fo troublefome; expectoration tinged with blood; appetite not fo bad; vertigo increafed, with the appearance of objects floating before the eyes; b. t. and fk. natural; pain of the breaft not fo much; p. 90, and irregular. Aug. dos. ad gt. xvi. ter in die. = 23. A reftlefs night; vomited, and the vertigo is become diftreffing, with frequent faintnefs; cough the fame, but expectoration lefs, though flightly tinged with blood; appetite bad; fears fhe fhall not be able to go on with the medicine; no fweats; b. t. and fk. natural, p. 96. Is perfuaded to perfevere: Rep. dofis ut heri. = 24. A much better night; cough not fo troublefome, and expectoration far lefs copious; vertigo lefs, appetite better; has obferved that her urine is more copious, though before it was always fcanty, and depofited a lateritious fediment; b. t. and fk. natural, p. 100. Aug. dos. ad gt. xvii. ter in die. = 25. An indifferent night; cough troublefome; expectoration diminifhes, though ftill flightly tinged with blood; vertigo as yefterday; appetite rather better; no vomiting; b. t. and fk. natural, p. 96. Aug. dos. ad gt. xviii. ter in die. = 26. A tolerable night; cough nearly the fame; expectoration diminifhes; complains of vertigo, with frequent faintnefs, and fenfe of finking at the ftomach; appetite as yefterday; fweats have entirely left her; urine flows ftill more copioufly, and without fediment; b. t. and fk. natural, p. 96. Rep. dos. ut heri. = 27. A tolerable night; cough troublefome; expectoration as yefterday; vomited this morning; complains of great forenefs; vertigo the fame; appetite as before; b. t. and fk. natural, p. 96. Rep. dos. ut antea. = 28. A much better night, and feels better in every refpect; has not vomited; cough not fo troublefome; expectoration lefs, though confiderably tinged with blood; appetite better, and vertigo lefs; b. t. and fk. natural, p. 96. Aug. dos. ad gt. xix. ter in die. = 29. Continues much better; a good night; cough lefs; expectoration lefs tinged with blood; vertigo and faintnefs by no means fo confiderable; appetite better; b. t. and fk. natural, p. 90. Aug. dofis ad gt. xx. ter in die. = 30. Symptoms equally or more favourable than yefterday; p. 88, and unequal. Cont. dofis = Dec. 1. Was fo giddy that fhe fell on going up ftairs, and is fomewhat bruifed in confequence, otherwife is nearly as yefterday; expectoration has diminifhed nearly one half in quantity; vomitted this morning; p. has rifen to 100. Rep. dofis = 2. A reftlefs night; vomited much this morning; very giddy with dimnefs of fight and faintnefs; complains fhe can hardly keep life within within her; cough as yefterday; expectoration lefs tinged with blood; general forenefs; no appetite; b. t. and fk. natural, p. 88. Is perfuaded to continue = 3. A bad night; frequent naufea, but no vomiting; vertigo rather lefs, and appetite fomewhat better; cough and expectoration as yefterday; being fatigued by fitting up, is refting herfelf on bed; b. t. and fk. natural, p. 92. Cont. = 4. A better night than for fome time paft; cough not fo troublefome, and expectoration lefs, without tinge of blood; free from pain; appetite better; vertigo and faintnefs lefs; b. t. and fk. natural, p. 100. Rep. dofis = 5. A good night; cough lefs troublefome; expectoration as yefterday; free from pain; no vomiting; vertigo lefs, though the dimnefs of fight is confiderable; appetite better; b. t. and fk. natural, p. 90. Is perfuaded to increafe the dofe: Aug. dos. ad gt. xxi. ter in die = 6. A reftlefs night; cough troublefome, though expectoration lefs; frequent naufea, but no vomiting; vertigo increafed, vifion confufed, and objects appear of a yellow tinge; appetite little; b. t. and fk. natural, p. 96. Rep. dos. ut heri = 7. Rather a better night; vertigo and dimnefs of fight diftreffing; being fomewhat difcouraged in confequence, has omitted the dofe of the Tincture this morning; cough and expectoration as yefterday; appetite fomewhat better; b. t. and fk. natural, p. 96. Rep. dos. ut heri = 8. A tolerable night; has continued her medicine; is free from pain; vertigo lefs; cough much lefs, and expectoration diminifhed full two-thirds; b. t. and fk. natural, p. 96. Is encouraged to perfevere: Rep. dos. = 9. A good night, without being difturbed by the cough, though it is fomewhat troublefome this morning; expectoration rather more copious than yefterday, but free from tinge of blood; feels quite eafy, and better in every refpect; vertigo rather lefs; p. 100. Increafed the dofe this morning, of her own accord, to gt. xxii. Cont. = 10. A good night; cough and expectoration as yefterday; the latter flightly tinged with blood; appetite better; free from pain; b. t. and fk. natural, p. 96. Since taking the tincture, has obferved a tumor in the right axilla, of confiderable fize, and of many years ftanding, is totally removed. Aug. dos. ad gt. xxiii. ter in die. = 11. A good night, free from pain; vertigo and confufion of vifion lefs; appetite better; b. t. and fk. natural; p. 92. Aug. dos. ad gt. xxiv. ter in die. = 12. A reftlefs night; vomited confiderably this morning, and is very languid; vertigo and confufion of vifion diftreffing; general forenefs; appetite indifferent; cough more troublefome; expectoration rather more copious, and flightly tinged with blood; b. t. and fk. natural, p. 88. Rep. dofis. = 13. A better night; has not vomited; cough troublefome; expectoration nearly the fame; vertigo vertigo and confuſion of viſion diſtreſſing; appetite bad; b. t. and ſk. natural, p. 88. Aug. doſis ad gt. xxv. ter in die. = 14. A tolerable night; cough not ſo troubleſome, and expectoration much diminiſhed; vertigo, and confuſion of viſion; when looking at ſilver, it appears of a yellow colour; appetite better; b. t. and ſk. natural, p. 92. Rep. doſis. = 16. A reſtleſs night; vomited conſiderably; cough and expectoration nearly the ſame; appetite a little better; other ſymptoms as yeſterday; p. 100. Aug. dos. ad gt. xxvi. = 17. A very good night; no vomiting; feels better than uſual this morning; appetite better; cough not ſo troubleſome, and expectoration much diminiſhed, without tinge of blood, and of a lighter colour; b. t. and ſk. natural, p. 88. Aug. dos. ad gt. xxvii. ter in die. = 18. An indifferent night; vomited this morning; cough rather troubleſome, and expectoration rather increaſed, though without tinge of blood, and of a lighter colour; appetite not ſo good; vertigo and confuſion of viſion increaſed, but does not feel that ſinking at the ſtomach ſhe complained of ſome time ago; b. t. and ſk. natural, p. 94. Aug. dos. ad gt. xxviii. = 19. A poor night; vomited; cough troubleſome; expectoration leſs; appetite rather better; other ſymptoms the ſame; p. 96. Aug. dos. ad gt. xxix. ter in die. = 20. A good night; no vomiting; expectoration much leſs; p. 100. Aug. dos. ad gt. xxx. ter in die.——Having now arrived at what is conſidered a ſtandard doſe, my patient was pleaſed that ſhe had got to her journey's end, and that ſhe ſhould not be requeſted to increaſe the number of drops. She, however, continued their uſe for upwards of three weeks from the laſt report, and every day experienced a change for the better; the pulſe keeping up almoſt invariably to 100. Her nights became nearly undiſturbed, her appetite good, her ſtrength in a great meaſure reſtored, and the expectoration, which, as mentioned, amounting to a full pint, of a purulent nature, emitting an abominable fœtor, was reduced to a little more than a tea ſpoonful of pure mucus, and perfectly inoffenſive in ſmell. It was my wiſh that ſhe might have continued the medicine for ſome weeks longer; but being tired of medicine, and imagining herſelf ſo far reſtored as to be ſecure from a ſecond attack, ſhe declined having any thing to do with it; obſerving, it was time enough to have recourſe to the remedy when the diſeaſe ſhould demand it.—The reduction of the pulſe in this caſe was by no means remarkable, though on a few occaſions it was pretty conſiderable; a proof that the good effects of this medicine does not depend upon the retardation of the circulation. Though the ſtate of the pulſe was particularly attended to, it was impoſſible to be ſo extremely preciſe as Dr. Magennis was in the Caſe inſerted in your your laſt Number, on account of the patient reſiding at ſome diſtance from the town. No doubt, improvement may be derived from ſuch accuracy, where it can be attended to; and I ſhould have been happy to have obſerved it myſelf, had it been poſſible. In the Caſe tranſmitted by Dr. Magennis, the pulſe was generally much reduced towards evening, which may be owing to the ſyſtem being then more under the influence of the medicine from the ſucceſſive doſes taken in the day time. The concluſions drawn by the Doctor, from the internal uſe of this medicine, I preſume, will be allowed to be extremely juſt and highly beneficial. The preſent Caſe, however, proves (differently from that related by Dr. M.) that the Tinct. Digital. is *certainly diuretic*, and *gently aperient*. It appears that this medicine has a wonderful influence on the abſorbent ſyſtem, from the circumſtance of an indolent, ſcrophulous tumor, full as large as an hen's egg, in the axilla, being totally removed during its adminiſtration. Yet this fact is not ſufficient to warrant the concluſion, that it is on this principle we are to account for the modus operandi of Digitalis; becauſe there are other medicines (ſuch as mercury for inſtance) which are ſuppoſed to act particularly on the abſorbents, and are conſidered as injurious in phthiſis pulmonalis. But however ſatiſfactory it might be to trace the modus operandi, it is comparatively of little conſequence as long as we are acquainted with the remedy. I am,

Gentlemen,

Your humble ſervant,

JOHN PENKIVIL, Surgeon.

*Plymouth Dock,*
*Feb.* 16, 1800.

